# The Subfamily-Specific Interaction between Kv2.1 and Kv6.4 Subunits Is Determined by Interactions between the N- and C-termini

**DOI:** 10.1371/journal.pone.0098960

**Published:** 2014-06-05

**Authors:** Elke Bocksteins, Evy Mayeur, Abbi Van Tilborg, Glenn Regnier, Jean-Pierre Timmermans, Dirk J. Snyders

**Affiliations:** 1 Laboratory for Molecular Biophysics, Physiology and Pharmacology, Department of Biomedical Sciences, University of Antwerp, Antwerp, Belgium; 2 Laboratory of Cell Biology and Histology, Department of Veterinary Sciences, University of Antwerp, Antwerp, Belgium; University of Sydney, Australia

## Abstract

The “silent” voltage-gated potassium (KvS) channel subunit Kv6.4 does not form electrically functional homotetramers at the plasma membrane but assembles with Kv2.1 subunits, generating functional Kv2.1/Kv6.4 heterotetramers. The N-terminal T1 domain determines the subfamily-specific assembly of Kv1-4 subunits by preventing interactions between subunits that belong to different subfamilies. For Kv6.4, yeast-two-hybrid experiments showed an interaction of the Kv6.4 N-terminus with the Kv2.1 N-terminus, but unexpectedly also with the Kv3.1 N-terminus. We confirmed this interaction by Fluorescence Resonance Energy Transfer (FRET) and co-immunoprecipitation (co-IP) using N-terminal Kv3.1 and Kv6.4 fragments. However, full-length Kv3.1 and Kv6.4 subunits do not form heterotetramers at the plasma membrane. Therefore, additional interactions between the Kv6.4 and Kv2.1 subunits should be important in the Kv2.1/Kv6.4 subfamily-specificity. Using FRET and co-IP approaches with N- and C-terminal fragments we observed that the Kv6.4 C-terminus physically interacts with the Kv2.1 N-terminus but not with the Kv3.1 N-terminus. The N-terminal amino acid sequence CDD which is conserved between Kv2 and KvS subunits appeared to be a key determinant since charge reversals with arginine substitutions abolished the interaction between the N-terminus of Kv2.1 and the C-terminus of both Kv2.1 and Kv6.4. In addition, the Kv6.4(CKv3.1) chimera in which the C-terminus of Kv6.4 was replaced by the corresponding domain of Kv3.1, disrupted the assembly with Kv2.1. These results indicate that the subfamily-specific Kv2.1/Kv6.4 heterotetramerization is determined by interactions between Kv2.1 and Kv6.4 that involve both the N- and C-termini in which the conserved N-terminal CDD sequence plays a key role.

## Introduction

Based on sequence homology, eight *Shaker*-related subfamilies of voltage-gated potassium (Kv) channels have been identified: Kv1-Kv6 and Kv8-Kv9 [1]. Each α-subunit consists of six transmembrane segments (S1-S6) and cytoplasmic N- and C-termini. Four α-subunits assemble into a Kv channel in which S5-S6 form the K^+^ selective pore while S1-S4 constitute the voltage sensing domain (VSD) [2]. Members of the Kv1-Kv4 subfamilies form electrically functional channels at the plasma membrane (PM) in both homo- and heterotetrameric configurations within each subfamily. This subfamily-specific channel assembly is controlled by the N-terminal tetramerization domain T1 that facilitates the assembly of compatible α-subunits into possible homo- and heterotetrameric channels and prevents subunits belonging to different subfamilies from assembling [3]–[5]. However, cross-subfamily tetramerization is possible without the T1 domain since subunits lacking the T1 domain can also assemble into electrically functional channels at the PM, albeit less efficiently [6]–[8]. For example, deletion of the N-terminal domain of the Kv2.1 and Kv1.4 subunits resulted in the loss of subfamily-restricted co-assembly of those subunits [3].

Even though members of the Kv5, Kv6, Kv8 and Kv9 subfamilies possess all the typical hallmarks of a Kv α-subunit, they do not form electrically functional homotetrameric channels at the PM. This is due to the retention of these “silent” subunits (KvS) in the endoplasmic reticulum (ER) [9]. Nonetheless, KvS subunits form electrically functional heterotetramers with members of the Kv2 subfamily that traffic to the plasma membrane [10]. Heterotetrameric Kv2/KvS channels exhibit distinct biophysical properties compared to homotetrameric Kv2 channels, but the degree of modulation varies between KvS subunits. The KvS subunits change the current density, shift the voltage-dependence of activation and inactivation, change the gating kinetics and/or alter the pharmacological properties, as compared to homotetrameric Kv2 channels [9].

In addition to the Kv2/KvS interaction, several KvS subunits have been suggested to interact with members of the Kv3 subfamily as well, since Kv3.4 current density was reduced after co-expression with Kv8.1, Kv9.1 and Kv9.3 [11], [12]. Furthermore, yeast-two-hybrid (Y2H) analysis revealed an interaction of the N-termini of the Kv6.3, Kv6.4 and Kv8.2 subunits with the N-terminus of Kv3.1 [10]. However, there is no evidence of Kv3/KvS channels at the PM. This suggests that the subfamily-specific assembly of KvS and Kv2.1 subunits into electrically functional channels at the PM is not exclusively determined by the N-terminal T1 domain of KvS subunits. Our results indicate that the subfamily-specific Kv2.1/Kv6.4 tetramerization requires specific interactions between the N-terminus of Kv2.1 and the C-terminus of Kv6.4.

## Materials and Methods

### Molecular Biology

Human Kv constructs were cloned in the mammalian vector peGFP-N1 (Clontech, Palo Alto, CA, USA). The Kv6.4 construct in which the C-terminus was exchanged for that of Kv3.1 as well as the N- and C-terminal segment constructs were constructed by PCR amplification using the QuickChange Site-Directed Mutagenesis kit (Stratagene La Jolla, CA, USA) and mutant primers. N- and C-terminal-tagged CFP and YFP constructs were obtained by subcloning the channel subunits in the peCFP-C1 and peCFP-N1 (Clontech) and peYFP-C1 and peYFP-N1 (Clontech) vectors, respectively. HA epitope-tagged Kv subunits were generated by introducing a HA tag in the extracellular S1-S2 loop. The presence of the desired modification and the absence of unwanted mutations were confirmed by DNA sequencing.

### Electrophysiology

HEK293 cells were cultured in MEM supplemented with 10% fetal bovine serum, 1% penicillin-streptomycin and 1% nonessential amino acids under 5% CO_2_. HEK293 cultures at 70% confluency were (co-)transfected with the cDNA of the unlabeled Kv2.1 and (chimeric) Kv6.4 subunits and 0.5 µg GFP as a transfection marker, according to the lipofection method using Lipofectamine2000 (Invitrogen, San Diego, CA, USA). Cells were trypsinized 16 to 24 hours after transfection and used for electrophysiological analysis within 5 h.

Whole cell current recordings were made at room temperature (22–23°C) using an Axopatch-200B amplifier (Axon Instruments, Union City, CA, USA) and were low-pass filtered and sampled at 1–10 kHz with a Digidata 1200A data-acquisition system (Axon instruments). Command voltages were controlled and data were stored using the pClamp10 software (Axon Instruments). Patch pipettes were pulled with a laser puller P2000 (Sutter Instruments, Novato, CA, USA) from 1.2 mm borosilicate glass (World Precision Instruments, Sarasota, FL, USA) and heat polished. Cells were superfused continuously with an extracellular solution containing (in mM): 145 NaCl, 4 KCl, 1.8 CaCl_2_, 1 MgCl_2_, 10 HEPES, 10 glucose and adjusted to pH 7.35 with NaOH. The pipettes were filled with intracellular solution containing (in mM): 110 KCl, 5 K_4_BAPTA, 5 K_2_ATP, 1 MgCl_2_ and 10 HEPES with the pH adjusted to 7.2 using KOH. Junction potentials between the extracellular and intracellular solution were zeroed with the filled pipette in the bath solution before sealing the cells. Cells were excluded from analysis if the series resistance exceeded 3 MΩ after compensation to ensure that voltage errors did not exceed 5 mV.

### Pulse protocols and data analysis

Voltage protocols are shown in the figures. The voltage dependence of channel inactivation was fitted with a Boltzmann equation according to: y = 1/{1+exp[−(V−V_1/2_)/k]} in which *V* represents the voltage applied, *V_1/2_* the voltage at which 50% of the channels are inactivated and *k* the slope factor. Results are presented as means ± S.E. Statistical analysis was performed using Student's t test or Mann-Whitney U Rank Sum test. P values<0.05 were considered to be statistically significant.

### Fluorescence resonance energy transfer (FRET) analysis

FRET experiments were performed on HEK293 cells that were cultured on coverslips and transfected with the appropriate cDNA as described above. CFP- and YFP-tagged subunits were co-transfected in a 1∶2 cDNA ratio to ensure that all FRET donor molecules were paired with the FRET acceptor molecules. Cells were used for FRET analysis 48 hours after transfection.

The fluorescent emission light of CFP (donor dye) and YFP (acceptor dye) molecules was determined using the Zeiss CLSM 510 microscope equipped with an argon laser for the visualization and bleaching of the CFP (excitation 458 nm) and YFP (excitation 514 nm) fluorophores. FRET efficiencies were determined using the following standard equation: FRET efficiency  = (1−(f_Da_-f_background_)/(f_D_-f_background_))×(1/pairedDA). After excitation at 458 nm, the CFP emission signal was recorded in the 464–490 nm bandwidth in both the presence of YFP (f_DA_, fluorescence signal of donor in the presence of the acceptor) and in the absence of YFP (f_D_, fluorescence of donor only). To determine f_D_, the YFP acceptor molecule was bleached by 30 s full power excitation at 514 nm laser light. Both f_DA_ and f_D_ were corrected for the background signal (f_background_) by determining the emission light in the 464–490 nm bandwidth after additional bleaching of CFP with a 30 s full power 458 nm laser light exposure. The paired DA fraction was assumed to be 1 as the used cDNA ratio of CFP- and YFP-tagged constructs was chosen to minimize the fraction of unpaired donor. Because the FRET efficiency is underestimated if the paired DA fraction is <1, only cells with an YFP/CFP intensity ratio >1 determined before YFP bleach, have been included in FRET efficiency analysis. FRET efficiencies were determined from three or more independent transfections.

### Co-immunoprecipitation (co-IP)

HEK293 cells were cultured in 75 cm^2^ culture flasks and transfected with 10 µg of the appropriate CFP- and HA-tagged constructs in a 1∶1 ratio using the Lipofectamine reagent according to the manufacturer's instructions (Invitrogen). Cells were solubilized 48 hours post-transfection in a 1 x PBS buffer supplemented with 5 mM EDTA, 1% Triton X-100 and a complete protease inhibitor mixture (Roche Diagnostics). Precipitation of the protein complexes from the soluble cell fraction was performed with GFP antibodies (Abcam, Cambridge, UK) and Protein G Agarose beads (Roche Diagnostics) that were pre-blocked with 2% non-fat milk powder in PBS. The proteins were eluted by incubating the beads in NuPAGE LDS sample buffer (Invitrogen) for 15 min at 37 °C. Subsequently, the eluted protein complexes were separated on a 4–12% Bis-Tris SDS-PAGE gel (Invitrogen) and transferred to a polyvinylidine difluoride (PVDF) membrane (GE Healthcare, Buckinghamshire, UK). The blot was blocked with 5% non-fat milk powder in PBS. Immunoprecipitated proteins were detected by incubation of the blots with anti-HA IgG (Roche Diagnostics) followed by incubation with anti-rat IgG conjugated to horseradish peroxidase (GE Healthcare) and subsequent ECL detection (GE Healthcare).

## Results

### The N-terminus of Kv6.4 physically interacts with the Kv2.1 and Kv3.1 N-termini

**Figure 1:**
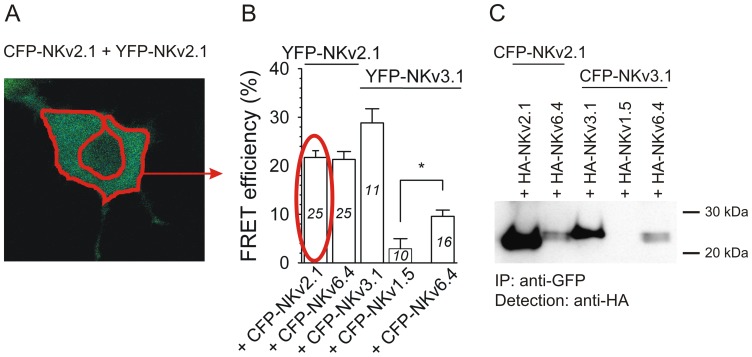
The N-terminal fragments of Kv3.1 and Kv6.4 physically interact. *A*, Representative cell expressing both CFP-NKv2.1 and YFP-NKv2.1. The red line encloses the region from which the FRET signals were determined. *B*, Average FRET efficiencies after co-expression of CFP- and YFP- labeled N-terminal Kv fragments. As positive controls YFP-NKv2.1+CFP-NKv2.1 (21.7±1.4%), YFP-NKv2.1+CFP-NKv6.4 (21.3±1.6%) and YFP-NKv3.1+CFP-NKv3.1 (28.8±2.9%) were used while the combination YFP-NKv3.1+CFP-NKv1.5 served as a negative control. Note the increased FRET efficiency after co-expression of the N-terminal Kv3.1 fragment (NKv3.1) with the N-terminal Kv6.4 fragment (NKv6.4) compared to the negative combination (9.6±1.5% and 2.9±2.1%, respectively). The numbers in each bar indicate the number of cells analyzed; *, p<0.05. *C*, Co-IP of CFP- and HA-tagged N-terminal Kv2.1, Kv6.4 and Kv3.1 fragments. Western blotting with a HA antibody after precipitation of the protein complexes from the soluble fraction with a GFP antibody demonstrated that the HA-tagged N-terminal fragment of Kv2.1 (HA-NKv2.1) and Kv3.1 (HA-NKv3.1) could be detected after co-expression with the CFP-tagged N-terminal Kv2.1 (CFP-NKv2.1) and Kv3.1 (CFP-NKv3.1) fragments (positive controls), respectively. In contrast, HA-NKv3.1 could not be detected upon co-expression with CFP-NKv1.5 (negative control). Note that – in addition to the expected interaction between the N-terminal Kv2.1 and Kv6.4 fragments (positive control) – HA-NKv6.4 could also be detected after co-expression with CFP-NKv3.1, indicating that the Kv3.1 and Kv6.4 N-termini physically interact.

In a previous study, a Y2H analysis revealed an interaction between the N-terminal fragments of the electrically silent subunit Kv6.4 and the electrically functional subunit Kv3.1 [10]. To confirm this interaction, we performed Fluorescence Resonance Energy Transfer (FRET, Fig. 1A–B) and co-immunoprecipitation (co-IP, Fig. 1C) experiments using the N-terminal Kv segments. Co-transfection of CFP- and YFP-tagged N-terminal Kv6.4 and Kv3.1 segments (CFP-NKv6.4 and YFP-NKv3.1, respectively) yielded a FRET efficiency of ∼10%. This FRET efficiency is lower than those observed with N-termini pairs that are known to form electrically functional channels at the PM (Fig. 1B; first 3 combinations), yet it is significantly higher than that obtained with the negative control (CFP-NKv1.5 + YFP-NKv3.1, ∼3%). Similar observations were obtained by co-IP experiments in which only the N-terminal segments were used. Figure 1C shows that the HA-tagged Kv6.4 N-terminal segment (HA-NKv6.4) could be clearly detected after precipitation of both the CFP-tagged Kv2.1 and Kv3.1 N-terminal segments (CFP-NKv2.1 and CFP-NKv3.1, respectively). These results are consistent with a physical interaction between the N-termini of Kv3.1 and Kv6.4.

### The Kv2.1 N-terminal and Kv6.4 C-terminal domains physically interact

Even though the N-termini of Kv3.1 and Kv6.4 can interact (as shown above), we have been unable to observe the formation of heterotetramers at the plasma membrane (Fig. S1). This suggests that additional interactions between the Kv6.4 and Kv2.1 subunits should be important in the subfamily-specific Kv2.1/Kv6.4 channel assembly. In the case of Kv2.1 homotetramers, it has been demonstrated that a physical interaction between the N- and C-termini is necessary for Kv2.1 functionality [13]. We hypothesized that similar interactions between the Kv2.1 and Kv6.4 N- and C-termini would also be responsible for the subfamily-specific formation of electrically functional Kv2.1/Kv6.4 channels at the PM.

**Figure 2:**
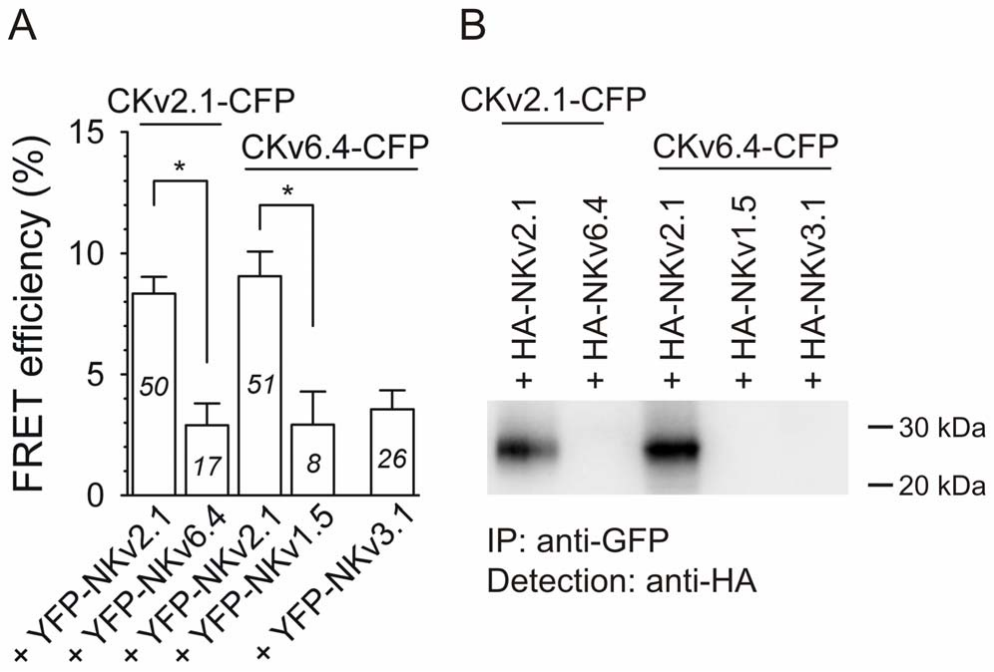
The Kv2.1 N-terminus physically interacts with both its own C-terminus and the Kv6.4 C-terminus. A, Average FRET efficiencies after co-expression of CFP- and YFP- labeled N- and C-terminal Kv fragments. As positive control, the previously described interaction between the Kv2.1 N- and C-terminus was used (8.3±0.7%) [13]. Note the significantly lower (*, p<0.05) FRET efficiency (2.9±0.9%) after co-expression of the CFP-tagged C-terminal Kv2.1 fragment (CKv2.1-CFP) with the YFP-tagged N-terminal Kv6.4 fragment (YFP-NKv6.4). In contrast, co-expression of YFP-NKv2.1 with CKv6.4-CFP yielded a significantly increased FRET efficiency compared to the negative YFP-NKv1.5+CKv6.4-CFP combination (8.7±1.3% and 2.9±1.4%, respectively, *, p<0.05). Note that the FRET efficiency of the CKv6.4-CFP+YFP-NKv3.1 combination (3.6±0.8%) was similar to the negative combination. *B*, Co-IP of CFP- and HA-tagged N- and C-terminal fragments. Immunoprecipitation was performed with a GFP antibody and Western blot was performed with a HA antibody. Note that the HA-tagged N-terminal Kv2.1 fragment could be detected after precipitation of both the C-terminal Kv2.1 and the C-terminal Kv6.4 fragment, while no interaction was observed after co-expression of CKv2.1-CFP with HA-NKv6.4 and CKv6.4-CFP with HA-NKv1.5 or HA-NKv3.1. This indicates that the Kv6.4 C-terminus physically interacts with the Kv2.1 N-terminus, but no interaction occurs between the Kv6.4 N-terminus and Kv2.1 C-terminus.

Co-expression of the CFP-tagged C-terminal segment of Kv2.1 (CKv2.1-CFP) with its YFP-tagged N-terminal segment (YFP-NKv2.1) yielded a significant FRET efficiency of ∼8% (Fig. 2A). These data confirmed the previously described physical interaction between the Kv2.1 N- and C-termini [13]. Co-expression of CKv2.1-CFP with the YFP-tagged N-terminal segment of Kv6.4 (YFP-NKv6.4) yielded a FRET efficiency of ∼3% which is similar to that observed for the incompatible CFP-NKv1.5 + YFP-NKv3.1 combination, suggesting that the Kv2.1 C-terminus does not interact with the N-terminus of Kv6.4. In contrast, co-expression of the CFP-tagged Kv6.4 C-terminus (CKv6.4-CFP) with YFP-NKv2.1 yielded a FRET efficiency of ∼9%, similar to that of the established CKv2.1-CFP + YFP-NKv2.1 interaction. These results suggest that only the N-terminus of Kv2.1 can interact with the C-terminus of Kv6.4 but not vice versa. These observations were further supported by co-IP experiments. The HA-tagged N-terminal domain of Kv2.1 could only be detected after precipitation of both the CFP-tagged Kv2.1 C-terminus and the CFP-tagged Kv6.4 C-terminus from the soluble fraction with a GFP antibody (Fig. 2B). No interactions were detected for other combinations of N- and C-termini. These results combined strongly suggest that the Kv2.1 N-terminus physically interacts with the C-terminus of both Kv2.1 and Kv6.4.

### The conserved N-terminal CDD sequence is an important determinant for the interaction between the Kv2.1 N-terminus and Kv6.4 C-terminus

We previously demonstrated that the negatively charged N-terminal CDD sequence (which is fully conserved in both the Kv2 and the KvS subfamilies but absent in the Kv1, Kv3 and Kv4 subfamilies) is involved in Kv2.1 and Kv2.1/Kv6.4 tetramerization. Charge reversal with arginine residues in full-length Kv2.1 reduced the assembly efficiency of Kv2.1(D74R,D75R) subunits into homotetrameric Kv2.1 channels. Furthermore, Kv6.4(D102R,D103R) subunits did not assemble into heterotetrameric channels with WT Kv2.1 [14]. This CDD sequence is within the N-terminal 17 amino acid motif that has been shown to interact with the 34 amino acid motif in the Kv2.1 C-terminus [13]. Therefore, we hypothesized that this CDD sequence at the N-terminus of Kv2.1 could also be a major determinant of the interaction with the C-terminus of Kv6.4.

**Figure 3:**
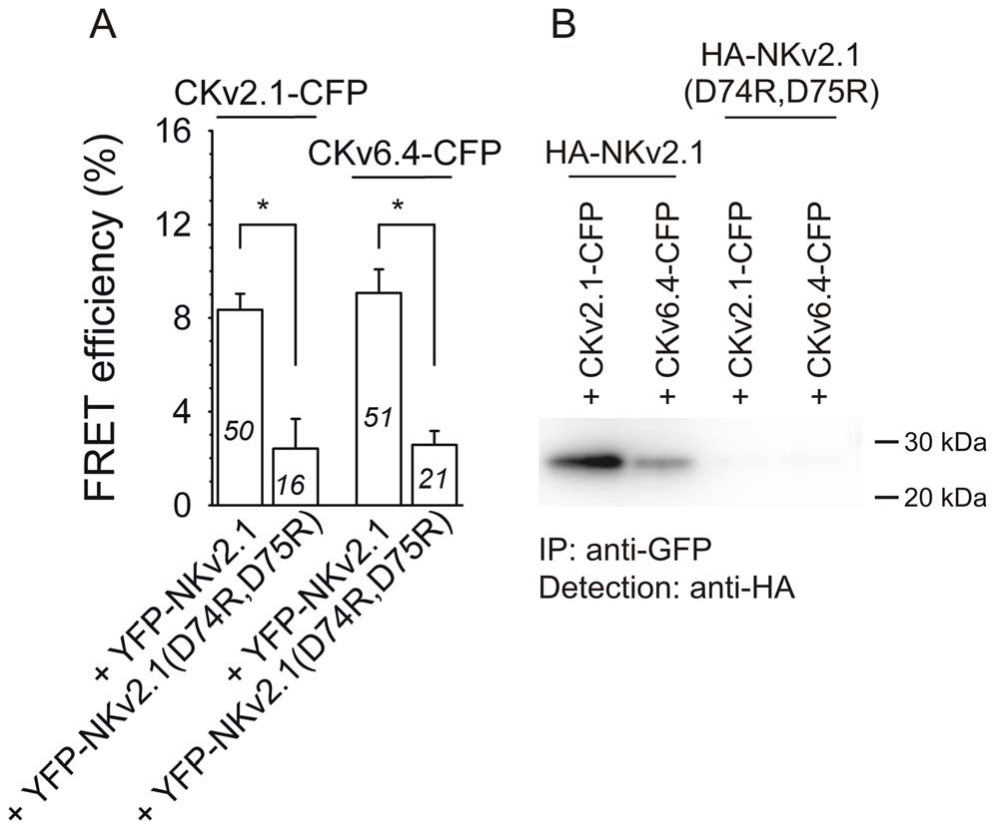
Mutating the conserved N-terminal CDD sequence disturbs the interaction between the Kv2.1 N-terminus and the Kv2.1 and Kv6.4 C-terminus. *A*, Average FRET efficiencies after co-expression of CFP- and YFP- labeled wild type and mutant N- and C-terminal Kv fragments. Note the significantly lower (*, p<0.05) FRET efficiency after co-expression of the YFP-tagged N-terminal Kv2.1 fragment in which the conserved CDD sequence has been mutated – YFP-NKv2.1(D74R,D75R) – with the CFP-tagged C-terminal Kv2.1 and Kv6.4 fragments (CKv2.1-CFP and CKv6.4-CFP, respectively) compared to the positive YFP-NKv2.1+CKv2.1-CFP and YFP-NK2.1+CKv6.4-CFP combinations (2.4±1.3%, 2.6±0.6%, 8.3±0.7% and 8.7±1.3%, respectively). *B*, Co-IP of CFP- and HA-tagged N- and C-terminal fragments. Immunoprecipitation was performed with a GFP antibody and Western blot was performed with a HA antibody. Note that the HA-tagged N-terminal Kv2.1 fragment could be detected after precipitation of both the C-terminal Kv2.1 and the C-terminal Kv6.4 fragment, while no interaction was observed after co-expression of HA-NKv2.1(D74R.D75R) with CKv2.1-CFP and CKv6.4-CFP, indicating that deletion of the conserved CDD sequence in the Kv2.1 N-terminus abolish the interaction between the Kv2.1 N-terminus and the Kv2.1 and Kv6.4 C-terminus.

To test our hypothesis, we first determined whether replacing the negatively charged aspartates of this CDD sequence by arginine residues disturbed the interaction between the Kv2.1 N-terminus and the C-termini of Kv2.1 and Kv6.4. FRET and co-IP experiments with the N-terminal segment of this Kv2.1(D74R,D75R) mutant –NKv2.1(D74R,D75R) – and the Kv2.1 and Kv6.4 C-terminal segments are shown in figure 3. Co-expression of YFP-NKv2.1(D74R,D75R) with the CFP-labeled Kv2.1 or Kv6.4 C-termini yielded FRET efficiencies that were significantly lower than those of the YFP-NKv2.1 + CKv2.1-CFP and YFP-NKv2.1 + CKv6.4-CFP combinations (Fig. 3A) suggesting that these mutations disrupted the interaction between the Kv2.1 N-terminus and the C-termini of Kv2.1 and Kv6.4. These results were confirmed by co-IP experiments (Fig. 3B); HA-NKv2.1(D74R,D75R) could not be detected after precipitation of the C-terminal Kv2.1 and Kv6.4 segments from the soluble fraction. Taken together, these results indicate that changing the conserved CDD sequence disrupts the physical interaction between the N-terminus of Kv2.1 and the C-termini of Kv2.1 and Kv6.4.

### Kv2.1/Kv6.4 heterotetramerization is disturbed when the C-terminus of Kv6.4 has been replaced with that of Kv3.1

**Figure 4:**
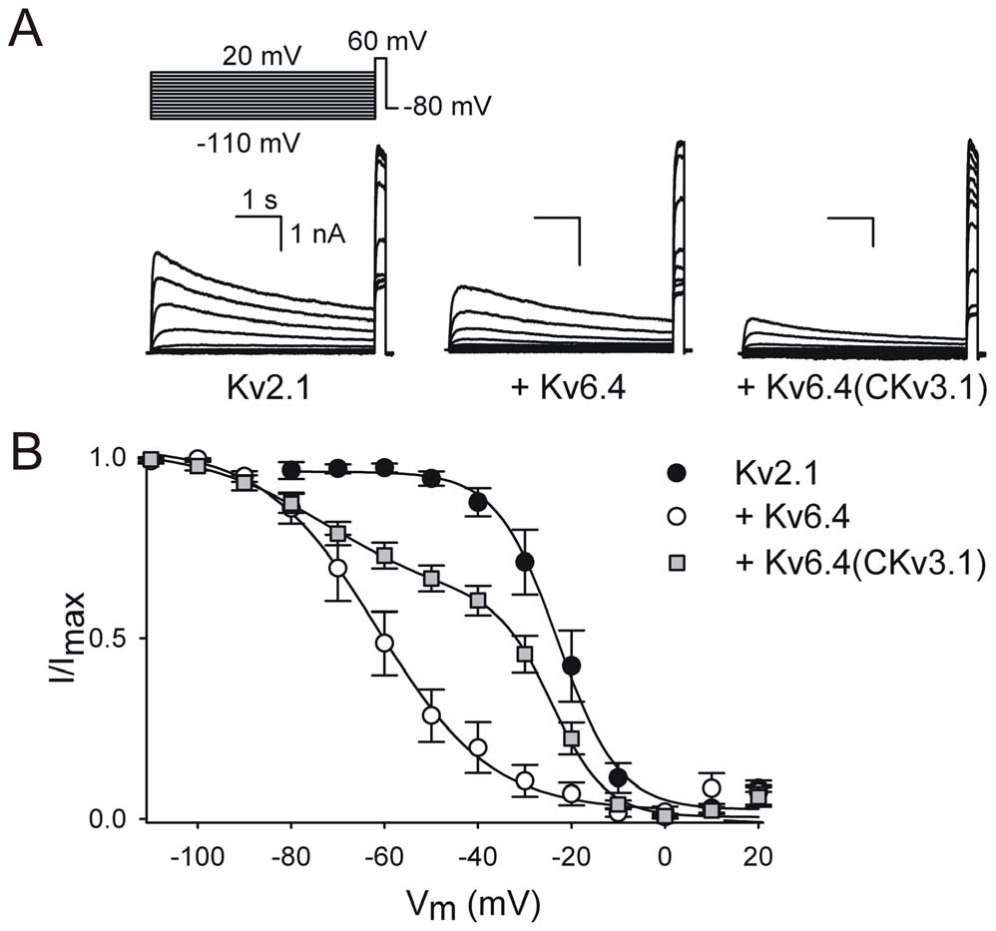
Replacing the Kv6.4 N- and/or C-terminus with the corresponding Kv3.1 fragments disturbs the interaction between Kv6.4 and Kv2.1. *A*, Typical whole-cell current recordings of Kv2.1 alone (left) and upon co-expression with Kv6.4 (middle) and Kv6.4(CKv3.1) (right) with the voltage protocol given on top. *B*, Voltage dependence of inactivation of Kv2.1 alone and upon co-expression with Kv6.4 and Kv6.4(CKv3.1). The inactivation curves were obtained by plotting the normalized peak current amplitude at +60 mV after a 5-sec prepulse as a function of the prepulse potential. Solid lines represent the (sum of two) Boltzmann fits. Co-expression with Kv6.4 shifts the voltage dependence of inactivation approximately 40 mV in the hyperpolarized direction compared to Kv2.1 alone. The voltage dependence of inactivation of the Kv2.1+Kv6.4(CKv3.1) combination was characterized by two components, suggesting the presence of a homotetrameric Kv2.1 and a heterotetrameric Kv2.1/Kv6.4(CKv3.1) channel population. This indicates that replacing the Kv6.4 C-terminus disturbs the Kv2.1/Kv6.4 channel assembly, suggesting that the Kv6.4 C-terminus is involved in the assembly of Kv2.1/Kv6.4 channels.

The results above suggest that the C-terminus of Kv6.4 and especially its interaction with the Kv2.1 N-terminus, is important in the subfamily-specific Kv2.1/Kv6.4 channel assembly. If this is the case, we would expect that altering the Kv6.4 C-terminus should also disturb the assembly of Kv2.1 and Kv6.4 into electrically functional Kv2.1/Kv6.4 heterotetramers at the PM. We investigated this using the chimeric Kv6.4(CKv3.1) construct in which the Kv6.4 C-terminus was replaced by the C-terminal domain of Kv3.1. Typical current recordings of Kv2.1 alone and upon co-expression with Kv6.4 and Kv6.4(CKv3.1) are shown in figure 4A. The main biophysical effect of WT Kv6.4 in a functional Kv2.1/Kv6.4 heterotetrameric channel is the approximately 40 mV hyperpolarizing shift in the voltage dependence of inactivation compared to Kv2.1 homotetramers. Indeed, the midpoint of inactivation for homotetrameric Kv2.1 currents was −23 mV (Fig 4B, filled circles and Table 1) which was shifted to −59 mV in heterotetrameric Kv2.1/Kv6.4 channels (Fig 4B, open circles and Table 1). Even though the ratio of Kv6.4 or Kv6.4(CKv3.1) DNAs to Kv2.1 DNA were the same, we consistently observed two components in the voltage dependence of inactivation upon co-expression of Kv2.1 with Kv6.4(CKv3.1) (Fig. 4B, grey squares and Table 1). One component has a midpoint of inactivation of −71 mV (n = 8) resembling the voltage dependence of inactivation of heterotetrameric Kv2.1/Kv6.4 channels (Table 1). The midpoint of inactivation of the second component was −24 mV, similar to that of homotetrameric Kv2.1 channels. The simplest explanation for these results is that co-expression of Kv2.1 with Kv6.4(CKv3.1) produces two populations of channels: a heterotetrameric Kv2.1/Kv6.4(CKv3.1) and a homotetrameric Kv2.1 channel population. This indicates that Kv6.4(CKv3.1) is still able to tetramerize with Kv2.1 although less efficiently as compared to wild type Kv6.4. These results support the notion that the C-terminus of Kv6.4 plays an important role in the subfamily-specific Kv2.1/Kv6.4 channel assembly.

**Table 1:** Voltage dependence of inactivation of Kv2.1 alone and upon co-expression with Kv6.4 and the Kv6.4(CKv3.1) chimera.

	Kv2.1			+Kv6.4			+Kv6.4(CKv3.1)		
**1^st^ component**									
*V_1/2_ (mV)*	−22.6	±	2.8		-		−24.3	±	1.7
*k*	5.9	±	0.5		-		4.9	±	0.3
**2^nd^ component**									
*V_1/2_ (mV)*		-		−59.3	±	4.6	−71.1	±	3.2
*k*		-		11.4	±	2.3	12.5	±	1.1
*n*		6			6			8	

The midpoints of inactivation (*V_1/2_*) and slope factors (*k*) were obtained from a single or double Boltzmann fit. Values are means ± S.E.; n, number of experiments; -, not applicable. For comparison, the parameters of Kv2.1/Kv6.4 channels are listed under the heading “2^nd^ component”.

## Discussion

Fully assembled Kv channels are tetramers of α-subunits. The subfamily-specific homo- and heterotetramerization of the Kv subunits belonging to the Kv1 through Kv4 subfamilies is controlled by the N-terminal T1 domain. An incompatible T1 domain prevents heterotetramerization between subunits of different subfamilies whereas a compatible T1 domain promotes the tetramerization of subunits from the same subfamily [3]–[5], [15]. This was supported by early observations that substitution of the N-terminal domain DRK1 (Kv2.1) with that of the *Shaker* B (ShB) subunit led to the assembly of the chimeric DRK1 subunit with ShB [16] and that deletion of the N-terminal domain of the Kv2.1 and Kv1.4 subunits resulted in the loss of subfamily-restricted co-assembly of those subunits [3]. Furthermore, specific residues of the T1 contact interface have been shown to be the key determinants for (subfamily-specific) Kv1-4 channel tetramerization [4], [17], [18]. It has been assumed that the subfamily-specific heterotetramerization between Kv2 and KvS subunits is governed by similar rules since specific residues in the T1 domain of both Kv2.1 and KvS subunits have been shown to be crucial for heterotetrameric Kv2/KvS channel assembly [14], [19]. However, several KvS subunits have been suggested to interact with members of the Kv3 subfamily [10]–[12]; Kv8.1, Kv9.1 and Kv9.3 reduced the Kv3.4 current [11], [12] and yeast-two-hybrid analysis revealed an interaction of the N-termini of Kv6.3, Kv6.4 and Kv8.2 with the N-terminus of Kv3.1 [10]. We confirmed the interaction between the Kv3.1 and Kv6.4 N-termini by Fluorescence Resonance Energy Transfer (FRET) and co-immunoprecipitation (co-IP) experiments (Fig. 1) but there is no evidence of Kv3.1/Kv6.4 channels at the PM (Fig. S1). This suggests that the subfamily-specific assembly of KvS and Kv2.1 subunits into electrically functional channels at the PM is not exclusively determined by the N-terminal T1 domain of KvS subunits.

**Figure 5:**
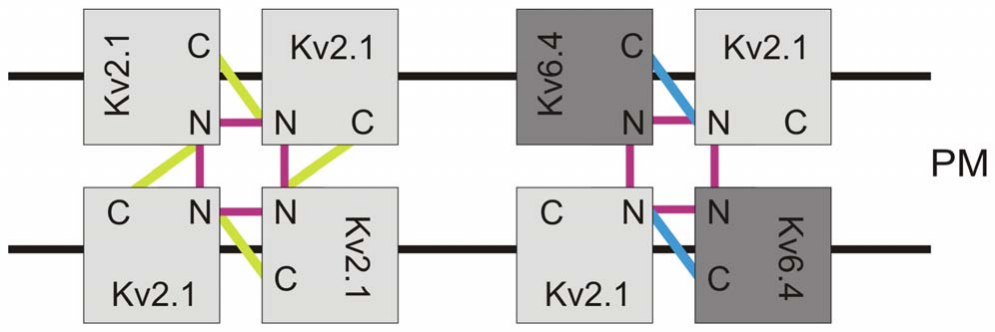
Schematic representation of the Kv2.1/Kv6.4 interactions. The interactions between the Kv6.4 (dark gray) and Kv2.1 (light gray) N-termini are presented in purple. The Kv2.1 N-terminus interacts with its own C-terminus (represented in yellow) and with the Kv6.4 C-terminus (represented in blue) allowing the formation of functional channels in the PM.

For Kv2.1, it has been suggested that the N-terminal T1 domain as well as the C-terminal domain play a role in channel assembly [20], [21]. Therefore, it is possible that the C-terminal domain is also involved in Kv2/KvS heterotetramerization. Our results demonstrate that the C-terminus of Kv6.4 interacted physically with the N-terminus of Kv2.1 but not with that of Kv3.1 (Fig. 2). Furthermore, replacing the Kv6.4 C-terminus with the corresponding Kv3.1 C-terminal domain was sufficient to disrupt the interaction of this chimeric Kv6.4(CKv3.1) subunit with Kv2.1 (Fig. 4). Taken together, these results indicate that the subfamily-specific Kv2.1/Kv6.4 channel assembly is determined by interactions between the Kv2.1 and Kv6.4 N- and C-termini, as represented in figure 5. In homotetrameric Kv2.1 channels, both N-terminal interactions (represented in purple in Fig. 5) and interactions between the Kv2.1 N- and C-termini (represented in yellow in Fig. 5) promote channel assembly. This is also the case in Kv2.1/Kv6.4 heterotetramers; interactions between the Kv2.1 and Kv6.4 N-termini (represented in purple in Fig. 5) as well as interactions between the Kv2.1 N-termini and the Kv6.4 C-termini (represented in blue in Fig. 5) promote the assembly of Kv2.1/Kv6.4 channels. For simplicity, only one possible Kv2.1/Kv6.4 stoichiometry (i.e. 2∶2) has been shown to represent the different interactions. However, a 3∶1 stoichiometry is also possible, as has been proposed for the interaction between Kv2.1 and Kv9.3 subunits into Kv2.1/Kv9.3 heterotetramers [22]. With such 3∶1 stoichiometry the sole interaction between the Kv6.4 C-terminus and the Kv2.1 N-terminus would promote Kv2.1/Kv6.4 channel assembly.

We previously demonstrated that the negatively charged CDD sequence which is fully conserved in both Kv2 and the KvS subfamilies, but absent in the Kv1, Kv3 and Kv4 subfamilies, is involved in Kv2.1/Kv6.4 tetramerization [14]. This CDD sequence is within the N-terminal 17 amino acid motif that has been shown to interact with the 34 amino acid motif in the Kv2.1 C-terminus [13]. Based on sequence homology, Kv6.4 and the other KvS subunits also possess this C-terminal 34 amino acid motif. Therefore, we hypothesized that this CDD sequence was the key determinant for the interaction between the N-terminus of Kv2.1 and the C-terminus of Kv2.1 and Kv6.4. Charge reversal arginine substitutions of this CDD sequence abolished the interaction between the Kv2.1 N-terminus and the C-terminus of both Kv2.1 and Kv6.4 (Fig. 4) indicating that this CDD sequence is an important determinant for the interaction between the N-terminus of Kv2.1 and the C-terminus of Kv6.4. In a homology model of the T1 domain of Kv2.1, this CDD sequence is located on a discrete loop at the bottom of this T1 domain [14] and this loop was the only striking difference between the Kv2.1 model and the available crystal structures of the T1 domain of Kv1.2 [23], Kv3.1 [17] and Kv4.2 [24]. It is conceivable that the 3D configuration of this conserved CDD sequence in the N-terminal domain of Kv2.1 is a key factor for the interaction between the N-terminus of Kv2.1 and the C-terminus of Kv2.1 and Kv6.4.

Our results demonstrate a physical interaction between the N- and C-terminal domains of Kv2.1 and Kv6.4 using soluble N- and C-terminal fragments. Due to greater restrictions on protein flexibility and accessibility in full-length channels, it may be possible that these interactions are different in fully assembled Kv2.1/Kv6.4 channels. However, interactions between N- and C-terminal domains have previously been demonstrated in several full-length ion channels. In the human ether-a-go-go related gene (hERG) channel, the interaction between the N-terminal Per-Arnt-Sim (PAS) domain and the C-terminal cyclic nucleotide-binding domain (CNBD) regulates the deactivating gating in hERG channels [25] while in cyclic nucleotide-gated (CNG) channels an interaction between the N-terminal domain and the C-terminal ligand-binding domain underlies the CNG channel activation [26]. Interactions between the N- and C-terminal segments have also been described in *Shaker* and in *Shaker*-related Kv channels. In *Shaker*, intracellular disulfide bond formation was detected under oxidizing conditions which was eliminated upon serine substitution of either the N-terminal or C-terminal cysteine residue [27]. For *Shaker*-related Kv channels it has been demonstrated that the Kv3.1 axon-dendrite targeting is controlled by an interaction of the axonal targeting motif in the Kv3.1 C-terminus, the Kv3.1 N-terminal T1 domain and the adaptor protein ankyrin G [28] while Kv2.1 requires a physical interaction between the N- and C-termini for proper functionality and channel assembly [13], [20], [21], [29]. In addition, our results showed that interactions between the N- and C-termini of Kv channels are also important to determine the subfamily-specificity of channel assembly.

In addition to the well-characterized interaction of KvS subunits with Kv2.1 subunits, previous studies have suggested that a number of KvS subunits interact with members of the Kv3 subfamily. This is based on the reduced Kv3.4 current density upon co-expression with Kv8.1, Kv9.1 and Kv9.3 [11], [12] and the reported interaction of the Kv6.3, Kv6.4 and Kv8.2 N-termini with the Kv3.1 N-terminus using the Y2H approach [10]. In this study, we demonstrated that a physical interaction does occur between the N-terminus of Kv3.1 and Kv6.4 (Fig. 1), but that this interaction is insufficient for the formation of heterotetrameric Kv3.1/Kv6.4 channels at the plasma membrane (Fig. S1). Indeed, such T1-T1 interactions could already occur while the growing polypeptide chains are still attached to the ribosomes and compatible T1 domains already associate while the transmembrane S1-S6 and C-terminal segments are still being processed within the ER translocator complex [30], [31].

Our results demonstrated that both N-N and N-C terminal interactions are needed to form electrically functional Kv2.1/Kv6.4 heterotetrameric channels at the plasma membrane. Furthermore, this N-C terminal interaction is supported by the conserved N-terminal CDD sequence in the Kv2 and KvS subunits. Therefore, we propose that this required interaction between the N-terminus of Kv2.1 and the C-terminus of Kv6.4 determines the subfamily-specific Kv2.1/Kv6.4 channel assembly.

## Supporting Information

Figure S1 **Full-length Kv3.1 and Kv6.4 subunits do not assemble into channels at the plasma membrane.** Visualization of membrane localized HA-tagged Kv6.4 subunits upon co-expression with Kv2.1-GFP (left) and Kv3.1-GFP (right) after staining transfected HEK293 cells with a HA antibody followed by an Alexa Fluor 546 antibody without permeabilizing the cells. Cells were fixed with 4% paraformaldehyde (PFA) 24 hours after transfection and incubated overnight with a rat anti-HA antibody (Roche Diagnostics, Basel, Switzerland) dissolved in a 0.1 M PBS solution containing 10% horse serum and 0.1% bovine serum albumin (BSA-c, Aurion, Wageningen, The Netherlands). Alexa Fluor 546 labeled anti-rat IgG (Invitrogen) in 0.1 M PBS +1% horse serum was used as secondary antibody (1∶1000) and incubated for 1 hour. Confocal images were obtained on a Zeiss CLSM 510 microscope equipped with an argon laser (excitation 458 nm) and a helium-neon laser (excitation 543 nm) for visualization of the GFP-tagged channels (emission signal recorded in the 500–550 nm bandwidth) and detection of the Alexa Fluor 546 antibody fluorescence (emission signal recorded beyond the 560 nm bandwidth), respectively. The subcellular localization of the (co)-expressed channels was determined in at least three independent experiments. Transfection of 5 µg Kv6.4-HA with 1 µg Kv2.1-GFP using the Lipofectamine reagent according to the manufacturer's instructions (Invitrogen, San Diego, CA, USA) resulted in a clustered membrane staining pattern that overlapped with the clustered Kv2.1-GFP membrane localization. In contrast, upon co-expression with 1 µg Kv3.1-GFP, no membrane staining originating from the Kv6.4-HA subunits could be detected, indicating that the Kv6.4 ER retention was not relieved. The top, middle and bottom panel in each column represent the fluorescence of the GFP-tagged channel subunit, the red fluorescence of the Alexa Fluor 546 antibody and the overlay of both, respectively. The yellow-brown color in the overlay indicates colocalization.
(TIF)
