# Supplementary figures and images for: The Subfamily-Specific Interaction between Kv2.1 and Kv6.4 Subunits Is Determined by Interactions between the N- and C-termini
Source: PLoS One. 2014 Jun 5;9(6):e98960. doi: 10.1371/journal.pone.0098960 (PMC4047056; doi:10.1371/journal.pone.0098960)

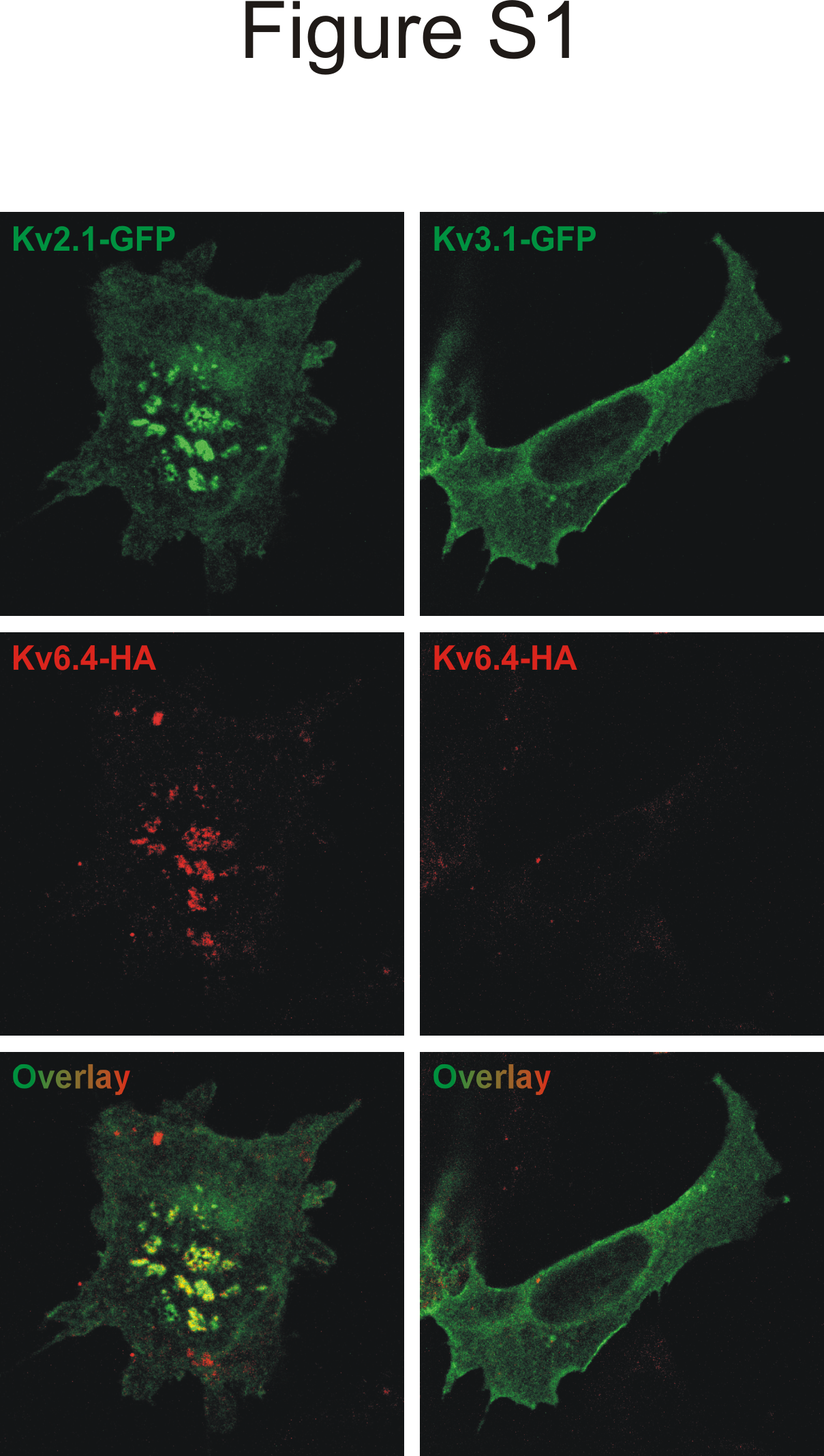

Supplement: Figure S1 — Full-length Kv3.1 and Kv6.4 subunits do not assemble into channels at the plasma membrane. Visualization of membrane localized HA-tagged Kv6.4 subunits upon co-expression with Kv2.1-GFP (left) and Kv3.1-GFP (right) after staining transfected HEK293 cells with a HA antibody followed by an Alexa Fluor 546 antibody without permeabilizing the cells. Cells were fixed with 4% paraformaldehyde (PFA) 24 hours after transfection and incubated overnight with a rat anti-HA antibody (Roche Diagnostics, Basel, Switzerland) dissolved in a 0.1 M PBS solution containing 10% horse serum and 0.1% bovine serum albumin (BSA-c, Aurion, Wageningen, The Netherlands). Alexa Fluor 546 labeled anti-rat IgG (Invitrogen) in 0.1 M PBS +1% horse serum was used as secondary antibody (1∶1000) and incubated for 1 hour. Confocal images were obtained on a Zeiss CLSM 510 microscope equipped with an argon laser (excitation 458 nm) and a helium-neon laser (excitation 543 nm) for visualization of the GFP-tagged channels (emission signal recorded in the 500–550 nm bandwidth) and detection of the Alexa Fluor 546 antibody fluorescence (emission signal recorded beyond the 560 nm bandwidth), respectively. The subcellular localization of the (co)-expressed channels was determined in at least three independent experiments. Transfection of 5 µg Kv6.4-HA with 1 µg Kv2.1-GFP using the Lipofectamine reagent according to the manufacturer's instructions (Invitrogen, San Diego, CA, USA) resulted in a clustered membrane staining pattern that overlapped with the clustered Kv2.1-GFP membrane localization. In contrast, upon co-expression with 1 µg Kv3.1-GFP, no membrane staining originating from the Kv6.4-HA subunits could be detected, indicating that the Kv6.4 ER retention was not relieved. The top, middle and bottom panel in each column represent the fluorescence of the GFP-tagged channel subunit, the red fluorescence of the Alexa Fluor 546 antibody and the overlay of both, respectively. The yellow-brown color in the ov [file pone.0098960.s001.tif]
